# Distinct dual roles of *p*-Tyr42 RhoA GTPase in tau phosphorylation and ATP citrate lyase activation upon different Aβ concentrations

**DOI:** 10.1016/j.redox.2020.101446

**Published:** 2020-01-31

**Authors:** Kim Cuong Cap, Yeon-Joo Jung, Bo Young Choi, Seung Jae Hyeon, Jae-Gyu Kim, Jung-Ki Min, Rokibul Islam, Abu Jubayer Hossain, Won-Suk Chung, Sang Won Suh, Hoon Ryu, Jae-Bong Park

**Affiliations:** a Department of Biochemistry, Hallym University College of Medicine, Chuncheon, Kangwon-do, 24252, Republic of Korea; b Department of Biological Science, Korea Advanced Institute of Science and Technology, Daejeon, 34141, Republic of Korea; c Department of Physiology, Hallym University College of Medicine, Chuncheon, Kangwon-do, 24252, Republic of Korea; d Laboratory for Brain Gene Regulation and Epigenetics, Center for Neuromedicine, Brain Science Institute, Korea Institute of Science and Technology, Seoul, 02792, Republic of Korea; e Institute of Cell Differentiation and Aging, Hallym University College of Medicine, Chuncheon, Kangwon-do, 24252, Republic of Korea; f Hallym Clinical and Translational Science Institute, Hallym University College of Medicine, Chuncheon, Kangwon-do, 24252, Republic of Korea; g Institute of Research and Development, Duy Tan University, Danang, 550000, Viet Nam; h Department of Biotechnology and Genetic Engineering, Faculty of Biological Science, Islamic University, Kushtia, 7003, Bangladesh; i eLmed Co., Hallym University College of Medicine, Chuncheon, Kangwon-do, 24252, Republic of Korea

**Keywords:** ACL, Alzheimer disease, Aβ, NADK, Superoxide, *p*-Tyr42 RhoA, p-Tau, 8-oxoG, 8-oxoguanine, Aβ, amyloid beta peptide, ACL, ATP citrate lyase, AD, Alzheimer disease, DPI, diphenyleneiodonium chloride, GSK-3β, glycogen synthase kinase-3β, GFAP, glial fibrillary acidic protein (astrocyte marker), Iba1, ionized calcium-binding adapter molecule 1 (microglia marker), NADK, NAD kinase, NeuN, neuronal nuclei specific antibody, NGF, nerve growth factor, ROCK, Rho-dependent coiled-coil kinase, ROS, reactive oxygen species, SREBP, sterol regulatory element-binding protein, mSREBP, matured SREBP

## Abstract

Both the accumulation of Amyloid-β (Aβ) in plaques and phosphorylation of Tau protein (p-Tau) in neurofibrillary tangles have been identified as two major symptomatic features of Alzheimer's disease (AD). Despite of critical role of Aβ and p-Tau in AD progress, the interconnection of signalling pathways that Aβ induces p-Tau remains elusive. Herein, we observed that a popular AD model mouse (APP/PS1) and Aβ-injected mouse showed an increase in *p*-Tyr42 Rho in hippocampus of brain. Low concentrations of Aβ (1 μM) induced RhoA-mediated Ser422 phosphorylation of Tau protein (*p*-Ser422 Tau), but reduced the expression of ATP citrate lyase (ACL) in the HT22 hippocampal neuronal cell line. In contrast, high concentrations of Aβ (10 μM) along with high levels of superoxide production remarkably attenuated accumulation of *p*-Ser422 Tau, but augmented ACL expression and activated sterol regulatory element-binding protein 1 (SREBP1), leading to cellular senescence. Notably, a high concentration of Aβ (10 μM) induced nuclear localization of *p*-Tyr42 Rho, which positively regulated NAD kinase (NADK) expression by binding to the *NADK* promoter. Furthermore, severe AD patient brain showed high *p*-Tyr42 Rho levels. Collectively, our findings indicate that both high and low concentrations of Aβ are detrimental to neurons *via* distinct two *p*-Tyr42 RhoA-mediated signalling pathways in Ser422 phosphorylation of Tau and ACL expression.

## Highlights

• Aβ stimulates at least two signalling pathways depending on its concentrations.
• *p*-Tyr42 RhoA is critical for the two signalling pathways.
• Low concentration of Aβ induces *p*-Ser422 Tau.
• High concentration of Aβ induces ACL expression along with highly producing superoxide.
• In particular, *p*-Tyr42 RhoA translocalizes to nucleus, where it regulates expression of NAD kinase (*NADK*).

## Graphical abstract

**Figure:**
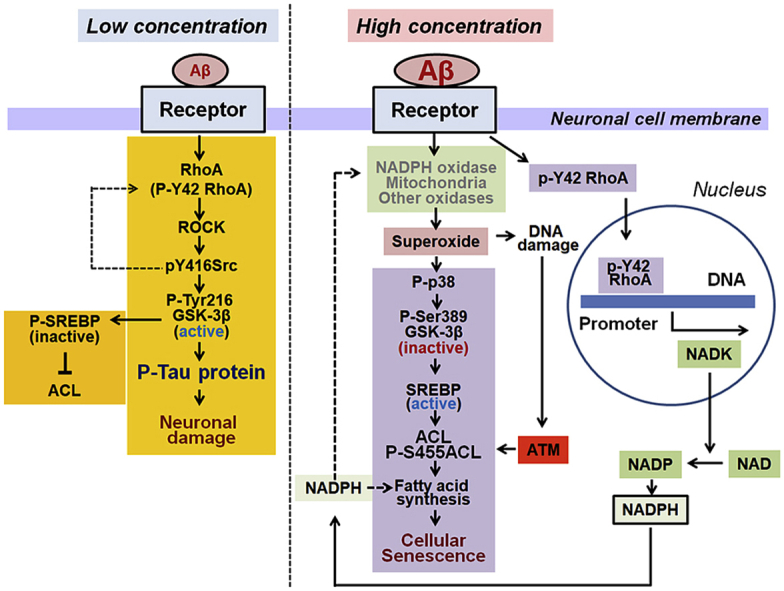

## 1 Introduction

Alzheimer's disease (AD), an age-related neurodegenerative disorder, is a major cause of dementia. The major pathological features of AD include extracellular senile plaques, consisting of amyloid-β (Aβ) peptide and intracellular neurofibrillary tangles (NFTs) consisting of aggregated hyperphosphorylated Tau protein. Notably, the accumulation of oligomeric and fibrillary Aβ contributes to hyperphosphorylation of the Tau protein through a series of neuronal signal transduction events [1]. Hyperphosphorylated Tau is resistant to different Tau proteases, leading to accumulation of Tau as NETs in neuron [2]. However, underlying mechanism by which Aβ induces Tau phosphorylation has not been well understood.

Tau is a microtubule (MT) associated protein and involved in the stabilization of axonal MT. The abnormal phosphorylation of Tau releases itself from MT, promoting disassembly and disruption of MT, leading to impairment of axonal MT function and ultimately to neuronal cell death. Tau proteins can be phosphorylated on at least 30 sites. Soluble Tau has a low ratio of phosphate/Tau, whereas paired helical filament Tau (PHF-Tau) has a much higher ratio of phosphate/Tau in AD brains [3]. The degree of phosphorylated Tau is attributed to the activities of protein kinases and phosphatases. Some kinases of two different types phosphorylate at least 30 serine/threonine residues of Tau: proline-directed kinases including MAPK, CDK2, CDK5, GSK-3, JNK/SAQPK, and p38, and non-proline directed kinases including PKA, CaMKII, and CKI/II [1].

Among them, GSK-3β is a well-known kinase responsible for hyperphosphorylation of the Tau protein. Two GSK-3 isoforms, GSK-3α (51 kDa) and GSK-3β (47 kDa), which are expressed in mammals, are constitutively active kinases without stimulation. GSK-3 is inhibited in response to two main activating stimuli, specifically the insulin and Wnt signalling pathways. Main substrates of GSK-3β are glycogen synthase (GS), β-catenin, and Tau protein [4,5]. GSK-3β can phosphorylate at least 15 residues of Tau protein: Ser46, Thr50, Thr175, Thr181, Ser199, Ser202, Thr205, Thr212, Thr217, Thr231, Ser235, Ser396, Ser404, and Ser413 [6]. *p*-Tyr216 GSK-3β is an active form, while *p*-Ser9 and *p*-Ser389 (in mouse or *p*-Thr390 in human) of GSK-3β are inactive forms. In fact, *p*-Tyr216 GSK-3β was found in the brain of AD patients [7]. However, the involvement of *p*-Tyr216 GSK-3β in phosphorylation of Tau has not been well established.

RhoA was first studied as a critical regulator of actin filament dynamics, and engaged in cellular regulation including cell morphology, migration, and transcription. Rho GTPases are activated by guanine nucleotide exchange factors (GEFs), leading to a GTP-bound form, and inactivated by GTPase activating proteins (GAPs), leading to a GDP-bound form [8]. Active RhoA-GTP binds to several effector proteins such as Rho-associated coiled-coil kinase (ROCK) to transmit signals to downstream components [9]. In particular, Aβ was reported to activate RhoA/ROCK, leading to activation of NADPH oxidase and subsequently generation of ROS in BV2 cells [10]. In particular, RhoA activation generally interferes with neurite out growth and neuronal differentiation while activation of Cdc42 and Rac1 induce neurite processes [11]. Lots of evidence revealed that dysregulated Rho GTPase activities are related to Aβ production and synaptic plasticity in AD [12]. Remarkably, hydrogen peroxide induces Tyr42 phosphorylation of RhoA, leading to NF-κB activation in cancer cell and tumor progression [13]. However, *p*-Tyr42 RhoA engagement in AD and regulatory function of RhoA on p-Tau in response to Aβ has not been clearly investigated.

In this study, we examined whether RhoA regulates the phosphorylation of Tau upon low and high concentration of Aβ. Thereby, we found that there are at least two signalling pathways by which low concentrations of Aβ activate the RhoA/ROCK/GSK-3β signalling pathway, leading to *p*-Ser422 Tau in the HT22 hippocampal cell line, and high concentrations of Aβ induce expression of ATP citrate lyase (ACL). In addition, we found that *p*-Tyr42 RhoA exists in nucleus, where *p*-Tyr42 RhoA regulates specific gene expression such as *NAD kinase* (*NADK*), which converts NAD to NADP.

## 2 Materials and methods

### 2.1 Materials

Tat-C3 fusion protein (Tat-peptide and C3 toxin) was purified from *Escherichia coli* BL-21 [14]. Y27632 (SCM075) was obtained from Millipore Sigma (Burlington, USA). LiCl (L4408), N-acetyl-l-cysteine (NAC, A7250), phosphatase inhibitor cocktail (P0044), cerulenin (C2389), DPI (D2926), LPS (*E. coli* 055: B5) and apocynin (A10809) were purchased from Sigma-Aldrich (St. Louis, USA). NGF 2.5S (1933065), fetal bovine serum (FBS, 12484010), Dulbecco's modified eagle's medium (DMEM, 11965092), and penicillin-streptomycin antibiotics (15140122) were from GibcoBRL (New York, USA). Protease inhibitor cocktail (K1007) was also purchased from ApexBio (Boston, USA). NSC 23766 (553502) was purchased from Calbiochem (La Jolla, USA). Mito-TEMPO (ALX-430-150) was from Enzo Life Science (New York, USA). Amyloid-β peptide (Aβ_1-42_: DAEFRHDSGYEVHHQKLVFFAEDVGSNKGAIIGLMVGGVVIA) was purchased from Sigma-Aldrich (St. Louis, USA). Short amyloid-β peptide (Aβ_25-35_: GSNKGAIIGLM) was obtained from A&PEP corporation (Chungnam, Korea). The peptides Aβ_1-42_ and Aβ_25-35_ were dissolved in 0.4 mM DMSO at a concentration of 1 M. Stock solution of Aβ (1 M) was diluted in 1 × PBS at a concentration of 5 mM. Stocks were aliquoted and incubated at 37 °C for 3 days to form aggregated Aβ peptides (fAβ) [15,16]. Anti-ATP citrate lyase (ACL, sc-517267), -p-S404 tau (sc-12952), -p21 (sc-397), -lamin B (sc-365962), -actin (sc-58673) and -tubulin (sc-32293) antibodies were purchased from Santa Cruz Biotechnology (Texas, USA). Anti-p-Y216 GSK3β (ab75745), -p-S422 Tau (ab79415) and -mSREBP1 (ab28481) antibodies were purchased from Abcam (Cambridge, UK). Anti-p-T180/Y182 p38 MAPK (9215), -p-T390 GSK3β (3548), -*p*-ATM (4526), -p-H2AX (2577) and -p16 (80772) antibodies were from Cell Signalling Technology (Danvers, USA). Anti-p-S9 GSK3β antibody (CSB-PA 166926) was from Cusabio Technology (Houston, USA). Anti-p-S389 GSK3β (14850-1-AP) was obtained Proteintech (Manchester, UK).

### 2.2 Cell culture

Cells were cultured in DMEM complemented with 10% heat-inactivated FBS for PC12, LN18 and HEK293 cells or 5% FBS for HT22 and BV2, plus 1% antibiotics (penicillin and streptomycin) at 37 °C in a humidified incubator with 5% CO_2_.

### 2.3 Animals

Animal care and experiment were performed under approval (KA2016-08) from the Institutional Animal Care and Use Committee of KAIST. Male APP/PS1 mice [B6C3-Tg (APPswe, PSEN1dE9)85Dbo, The Jackson Laboratory] were bred with female C57BL/6 mice and maintained under constant temperature, humidity, and 12 h-light/12 h-dark cycle. C57BL/6 mice were purchased from Samtako (Samtako Inc. South Korea). Heterozygous APP/PS1 mice were genotyped by primers for PSEN1 (transgene 608 bp, and internal positive control 324 bp) and APP (377 bp). APP/PS1 male mice of 4–5 generation without apparent seizure were used. A few amyloid plaques were started to develop in the cerebral cortex at the age of 4–6 months of APP/PS1 transgenic mice by Thioflavin S (ThS) staining. The experiments were performed on wild-type and APP/PS1 (C57BL/6 mice) at 9-months-old (3 male mice), 12-months-old (3 male mice), and 16-months-old (3 male mice). In addition, C57BL/6J mice (4 months, male) were used for Aβ injection to hippocampal region.

### 2.4 Preparation of mouse brain tissues and immunocytochemistry and imaging of mouse brain tissues

All mice were anesthetized with avertin and were perfused with PBS followed by 4% paraformaldehyde (PFA). The brains were post-fixed in 4% PFA at 4 °C overnight and placed in 30% sucrose before freezing. Serial 10-μm coronal sections of the brain were collected for immunohistochemistry. The sections were blocked with blocking buffer (4% BSA and 0.3% Triton X-100 in DPBS) for 1 h at room temperature and then incubated with primary antibodies at 4 °C overnight. Primary antibodies were diluted as follows; anti-phospho RhoA (Y42) (rabbit polyclonal, 1:500), anti-NeuN (mouse monoclonal, 1:500, Millipore), anti-MAP2 (chicken polyclonal, 1:1,000, Abcam), anti-6E10 (mouse monoclonal, 1:1,000, Biolegend), anti-GFP (chicken polyclonal, 1:1000, AVES), anti-VGLUT1(guinea pig polyclonal, 1:1000, Millipore), anti-AIF/Iba-1 (goat polyclonal, 1:500, Novus), anti-GFAP (chicken polyclonal, 1:1000, AVES) and anti-TAU (phospho S422) (rabbit monoclonal, 1:500, Abcam). The tissues were washed five times with PBST (0.1% Tween 20) and then, incubated in secondary antibodies for 2 h at room temperature before mounting with vectashield (Vectorshield, Vector Lab.). Immunofluorescence images were acquired using a LSM 880 confocal microscope (Carl Zeiss) and then and then confocal images were taken by Z-stack images with the same intervals. For CA1 and SLM region, image stacks were obtained using a 20x objective by a 1024× 1024 pixel and the acquired images were represented with Fiji program (Image J, NIH, USA). Generally, it is well known that anti- MAP-2, NeuN, anti-GFAP, and anti-AIF/Iba-1 are specific marker for neuron, astrocyte and microglia in previous articles. In addition, we have tried to confirm that the pattern of immunostaining in various primary neurons, and glia as well as mouse brain tissues were similar with previous articles.

### 2.5 Preparation of anti-phospho-Tyr42 Rho antibody

The anti-*p*-Tyr42 Rho antibody was produced by Young-In Frontier (Seoul, Korea). Briefly, *p*-Tyr42 Rho peptide (epitope peptide T^37^VFEN(phospho-)Y^42^VADIE^47^) was synthesized using phospho-Tyr42 precursor. Fluorenylmethyloxycarbonyl (Fmoc)-Tyr(PO(Nme_2_)_2_)-OH was used as a precursor amino acid derivative to protect phosphate of Tyr42 from reaction and the protective groups (N(Me_2_)_2_) were removed after synthesis of peptide containing *p*-Tyr42. The peptide was purified by using C18 column and confirmed with Mass analysis. The peptide was conjugated to BSA, which was injected into rabbit to produce polyclonal anti-*p*-Tyr42 Rho antibodies. The serum containing antibody was purified through three steps: proteinA-bead, non-phospho peptide-bead to exclude dephospho-Rho antibody, and then *p*-Tyr42 peptide-beads were exploited to purify specific *p*-Tyr42 Rho GTPase antibody. Polyclonal anti-phospho Tytr42 Rho antibody revealed the specificity for *p*-Tyr42 RhoA [13]. In addition, RhoA antibody recognized RhoA in control cells while *p*-Tyr42 Rho antibody revealed the signal in only stimulated cells.

### 2.6 GTP-RhoA pull-down assay

Cells were maintained in DMEM without serum for 12 h followed by stimulation with appropriate concentration of Aβ. Stimulated cells washed twice with 1 × PBS were lysed in lysis buffer A (25 mM Tris pH 7.5, 5 mM MgCl2, 150 mM NaCl, 5% glycerol, and 1% NP-40) containing 1% phosphatase and 1% protease inhibitor cocktail. Cell lysates refined by centrifugation were equalized for total volume and protein concentration then incubated with GST-Rhotekin-Rho-binding domain beads for 3 h at 4 °C. The bound fractions (active RhoA-GTP and GST-RBD beads) were thrice washed with ice-cold lysis buffer B (50 mM Tris pH 7.4, 0.5 mM MgCl2, 150 mM NaCl, and 1% Triton X-100) containing 1% phosphatase inhibitor cocktail and 1% protease inhibitor cocktail, and separated on SDS-PAGE. A reserved aliquot of whole cell lysate was used to analyse total RhoA levels. Active RhoA was determined by measuring RhoA associated with GST-RBD beads with western blotting. The relative population of active RhoA was quantified by taking the ratio of active RhoA divided by total RhoA.

### 2.7 si-RNA transfection

Small interfering RNA (si-RNA) against RhoA (sc-36414), ATP citrate lyase (sc-45206), ATM (sc-29762) and control si-RNA (sc-37007) were purchased from Santa Cruz Biotechnology. The cells were seeded to 30%–40% confluency then transfected with si-RNAs using the X-tremeGENE siRNA transfection reagent (Cat. No. 04476093001 Sigma-Aldrich), according to the manufacturer's instructions. Briefly, 10 μl transfection reagent was added to 100 μl of serum-free medium containing 50 nM of each siRNA followed by incubation for 20 min at room temperature. The cells were incubated for 72 h and then protein expression was measured by Western blot analysis.

### 2.8 Superoxide measurement

Superoxide was directly measured in live cells using a dihydroethidium assay kit (Invitrogen: D11347). Briefly, cells (2 × 10^5^) were stimulated with proper concentrations of Aβ for various periods in serum-free medium, washed and fixed by 4% formaldehyde (15 min) at RT. To generate fluorescence, the cells were treated with 50 μM hydroethidine in DMSO (500 μl) for 15 min at RT, then washed with 1 × PBS two times. Fluorescence images were captured under a fluorescence microscope (Axiovert 200, Zeiss; Göttingen, Germany) with a filter of 540–552 nm for an excitation wavelength and with a filter of greater than 590 nm as an emission wavelength. IPLab 3.65α software was used to process images.

### 2.9 Assay for senescent-associated β-galactosidase activity

The senescence β-galactosidase (SA-β-gal) staining kit was designed to detect β-galactosidase activity of senescent cells according to the manufacturer's protocol. In brief, HT22 cells were seeded in a 6-well plate and treated with proper concentrations of Aβ. Cells were rinsed with 2 ml 1 × PBS and fixed for 15 min at RT with 1 ml fixative solution. After incubation with the staining solution overnight at 37 °C, slides were visible by microscope and scored for the SA-β-gal label as indicated by blue/green reactivation product over the cell soma using Photoshop cc2018 software (Adobe Inc.). SA-β-gal-positive cells were quantified by counting five random fields per slide. The ratio of the SA-β-gal-positive cells was obtained to estimate the degree of senescence-associated cells.

### 2.10 Cytosolic and nuclear fraction preparation of cells

Cytosolic and nuclear fractions were separated using NE-PER nuclear and cytoplasmic extraction reagents (CER: Thermo Scientific, 78833). Briefly, HT22 cells were stimulated with proper concentrations of Aβ, harvested in ice-cold 1 × PBS, pelleted by centrifuging at 13,000×*g* for 20 min. Fresh cell pellet (20 μl) was added to ice-cold CER I (200 μl), II (11 μl) plus protease inhibitors, vortexed and centrifuged on an appropriate setting to attain a cytoplasmic protein extract (the supernatant). Remaining pellets, which contain nuclei were suspended in ice-cold NER, vortexed and centrifuged to get the nuclear extract. Fractions were analysed by immunoblotting with proper antibodies and lamin B and tubulin proteins were used as a marker for nucleus and cytosol, respectively.

### 2.11 MTT cell proliferation inhibition assay

HT22 cells were seeded in 96-well plates at a density of 800 cells per well and incubated at 37 °C with pre-treatment of cerulein for 1 h. Different concentrations of Aβ and cerulenin were added in triplicate to the plates. The cells were incubated at 37 °C for 12–24 h and then 25 μl MTT (Sigma, USA) was added to each sample; after 4 h, 100 μl DMSO (Sigma, USA) was added to each well. The absorbance was measured at 570 nm, and the viability of the untreated cells was arbitrarily set at 100% compared with the viability of Aβ- or cerulenin-treated cells.

### 2.12 Western blotting

Cells rinsed in ice-cold 1 × PBS were harvested and lysed in RIPA buffer (50 mM Tris-HCl pH 7.5, 1 mM MgCl_2_, 1% Nonidet P-40, 150 mM NaCl) including 1% phosphatase/protease inhibitor cocktail. Cell lysates were centrifuged at 13,000×*g* for 20 min at 4 °C. Protein cell lysates (20–30 μg/lane) were loaded onto SDS-PAGE gels and then transferred to a PVDF membrane. Blots were probed with several antibodies. Protein bands were detected using enhanced chemiluminescence (ECL) and fusion FX system (Vilber Lourmat, France).

### 2.13 Human tissues and transcriptome analysis

Neuropathological processing of control and AD human brain samples was performed according to the procedures previously established for the Boston University Alzheimer's Disease Center (BUADC) and Chronic Traumatic Encephalopathy (CTE) Center. Institutional review board approval for ethical permission was obtained through the BUADC and CTE Center. Because the study involved only tissue collected from post-mortem individuals, which are not classified as human subjects, the Institutional Review Board approval was exempted. Next of kin provided informed consent for participation and brain donation. The study was performed in accordance with the institutional regulatory guidelines and principles of human subject protection in the Declaration of Helsinki. Detailed information about the brain tissues is described in Supplementary Table 1. In all cases in which AD was diagnosed at autopsy, AD was stated as the cause of death. Analysis of transcriptome of mRNA expression levels was performed using 6–9 tissue samples, which were obtained from temporal cortex brain of normal and AD patients.

### 2.14 Immunohistochemistry for the human brain tissue

#### 2.14.1 First staining

Paraffin-embedded tissues were sectioned in a coronal plane at 20 μm. The tissue sections were rehydrated, blocked with blocking solution [1% hydrogen peroxide (H_2_O_2_)], and incubated with rabbit polyclonal antibody to p-Y42 RhoA (1:200 dilution) and GSK3β-Y216 (1:200 dilution) for 24 h. After washing three times, the slides were processed with Vector ABC Kit (Vector Laboratories, Inc., Burlingame, CA, USA). The immunoreactive signals were developed with DAB chromogen (Thermo Fisher Scientific, Meridian, Rockford, IL, USA).

#### 2.14.2 Second staining

Endogenous alkaline phosphatase was blocked using 3% H_2_O_2_ in TBS. Sections were blocked with 2.5% normal horse serum (Vector Laboratories) before incubation for 24 h with a mouse monoclonal antibody to Aβ (1:200 dilution; BioLegend, San Diego, CA, USA). After washing, sections were incubated with ImmPRESS-AP anti-rabbit IgG (alkaline phosphatase) polymer detection reagent (Vector Laboratories) for 30 min at room temperature. Colors were developed with a Vector Red alkaline phosphatase substrate kit (Vector Laboratories). Slides were subsequently counterstained with hematoxylin (Vector Laboratories), and processed back to xylene through an increasing ethanol gradient [70%, 80% and 95% (1 × ), and 100% (2 × )] and then mounted. The images were analysed under a bright field microscope.

### 2.15 Cell morphology determination using coomasie-staining

Cells were seeded either in 6-well dishes (4 × 10^5^ cells/well). After treating with appropriate stimulation, cells were fixed by 4% paraformaldehyde at room temperature for 15 min, stained with Coomassie Brilliant Blue R-250 (C2006) for 5 min, washed 3 times with PBS. Three images (30–50 cells/each) at different locations in each well were acquired on a TMS-F #211128 microscope (Nikon) equipped with a Nikon Digital D5100 camera. Each experiment was done 3 times. Mean neurite outgrowth length or cell size was quantified in Photoshop cc2018 software (Adobe Inc.) by applying a grid to the pictures and counting intersections of neurites with the grid lines and total cell bodies and calculating the ratio thereof.

### 2.16 Statistical analysis

The western blotting of protein bands shown and statistical significance was based on analysis done with Photoshop cc2018 software (Adobe Inc.) and Prism 8.0 software (GraphPad), respectively. Generally, the data were shown as the means ± SE of at least three independent experiments with one- or two-way ANOVA analysis; if not, the fold number of protein band on western blotting was denoted. Values of the cell image data are means of three independent experiments (30–50 cells/image and 3 images/experiment) ± SE, one- or two-way ANOVA. The fluorescence images of brain tissues were quantified from two confocal images/sample and two samples/mouse using 3 male mice and statistical analysis of significance was based on two-way ANOVA analysis (**p < 0.01; ***<0.001; ****<0.0001). Transcriptome of human brain samples were analysed by unaired, two-tailed *t*-test (p*<0.05).

## 3 Results

### 3.1 An increase of *p*-Tyr42 Rho GTPase is observed in the AD model mouse

**Fig. 1:**
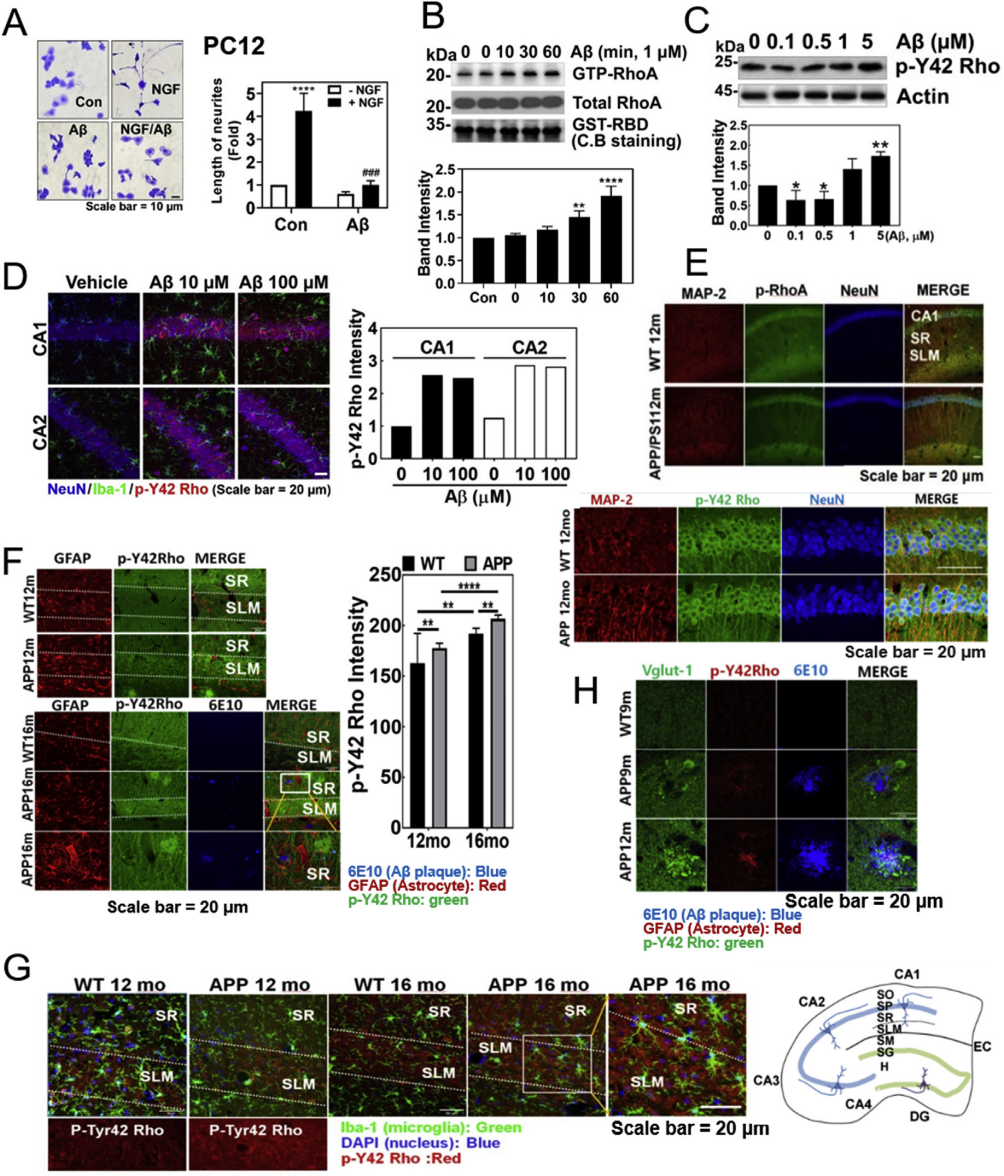
**P-Tyr42 Rho GTPase is increased in Aβ-induced neuronal cells and an AD model mouse. (A)** PC12 cells were treated with NGF (100 ng/ml, 72 h) with or without fAβ (1 μM, 24 h). The cells were stained by Coomassie-Blue and the length of neurite outgrowth was analysed using Photoshop CC2018 software (one-way ANOVA: p***<0.001, ****<0.0001). **(B)** GTP-RhoA levels of PC12 cells were measured using a pulldown assay with GST-RBD after fAβ (1 μM) treatment for the given times (one-way ANOVA: p**<0.01, ****<0.0001). **(C)** HT22 cells were exposed to the various Aβ concentrations for 24 h, and *p*-Tyr42 Rho (p-Y42 Rho) was detected by Western blot. (one-way ANOVA: p*<0.05, ** < 0.01). **(D)** Immunofluorescence of a vehicle- and sAβ (10 μM or 100 μM)-injected CA1 and CA2 regions of C57BL/6 mice brains (4 months of age, male) was revealed with anti-NeuN (neuronal nucleus), -Iba-1 (microglia), and -p-Y42 Rho antibodies. **(E**–**H)** Immunofluorescence staining of the brain tissues sections of WT and APP/PS transgenic C57BL/6 mice brains (9 months: WT and APP/PS1, each 3 male; 12 months: WT and APP/PS1, 3 male; 16 months, WT and APP/PS1, each 3 males) was performed with MAP2 (neuron marker) Ab, NeuN (neuronal nucleus marker) Ab, Iba-1 Ab (microglia marker, green), DAPI (nucleus marker, blue), and p-Y42 Rho Ab, or GFAP Ab (astrocyte marker, red), 6E10 Ab (amyloid plaque marker, blue), Vglu-1 (vesicular glutamate receptor-1), and p-Y42 Rho antibodies. Three male mice (3 samples per mouse and 2 images per sample) were used and we presented the representative image of them and the representative images were shown. The intensity was quantified and compared using unpaired, two-tailed *t*-test (F, p**<0.01, ****<0.0001). As a reference, a diagram of the hippocampus is illustrated: CA1-4, sectors 1–4 of the cornu ammonis; DG, dentate gyrus; EC, entorhinal cortex; H, hilus; SL, stratum lucidum; SLM, stratum lacunosum moleculare; SM, stratum moleculare; SO, stratum oriens; SP, stratum pyramidale; SR, stratum radiatum (right panel of Fig. 1G). (For interpretation of the references to colour in this figure legend, the reader is referred to the Web version of this article.)

It has been well known that NGF induces neurite outgrowth from PC12 cells, but we found that Aβ markedly attenuated neurite outgrowth from PC12 cells even in the presence of NGF (Fig. 1A). Since activated RhoA has been reported to inhibit neuronal differentiation [11], we examined whether Aβ effects on RhoA activity, and found that Aβ treatment elevated RhoA-GTP levels in HT-22 cells, a mouse hippocampal cell line (Fig. 1B). Recently, we found that hydrogen peroxide up-regulates *p*-Tyr42 RhoA, leading to NF-κB activation and cancer cell proliferation. In addition, we developed a novel antibody to recognize the *p*-Tyr42 residue of Rho GTPase [13]. Here, we observed that Aβ induced an increase of *p*-Tyr42 Rho GTPase in HT22 cells (Fig. 1C). Thereby, we examined whether *p*-Tyr42 Rho is related to Aβ-mediated AD in model mice brain. Soluble Aβ was injected to the hippocampus of mouse brain and allowed to diffuse, which stimulated hippocampus for 24 h (sFig. 1). *p*-Tyr42 Rho was much increased in neurons of cornu ammonis 1 (CA1) and CA2 regions by 5 μl of 10 and 100 μM Aβ (Fig. 1D). However, the actual resultant Aβ concentration in hippocampus would be reduced by a large factor due to dilution of Aβ by diffusion. As another model of AD, we used APP/PS1 transgenic mice. We observed the immunoactivity of *p*-Tyr42 Rho in the CA1 region of hippocampus in 12 month-old APP/PS1 transgenic mice brain. P-Tyr42 Rho found to be in CA1 and stratum lacunosum moleculare (SLM) layers of neuron (Fig. 1E). In 12 and 16 months-old APP/PS1 transgenic mice, *p*-Tyr42 Rho was significantly increased in SLM layer of hippocampal region of brain compared to wild type. In addition, 16 months-old mice revealed more *p*-Tyr42 Rho than 12 months-old mice (Fig. 1F). *p*-Tyr42 Rho was co-localized with neither glial fibrillary acidic protein (GFAP: astrocyte marker) nor ionized calcium-binding molecule 1 (Iba1, also known to be allograft inflammatory factor 1 AIF-1: microglia marker), suggesting that *p*-Tyr42 Rho does not generally exist in glial cells (Fig. 1F and G). *p*-Tyr42 Rho was also found to be co-localized with some of vesicular glutamate transporter 1 (Vglut-1), a synaptic vesicle marker-positive neuron associated with Aβ plaque in the APP/PS1 mouse brain (Fig. 1H).

### 3.2 Low concentrations of Aβ induce Ser422 phosphorylation of tau protein through RhoA activation

**Fig. 2:**
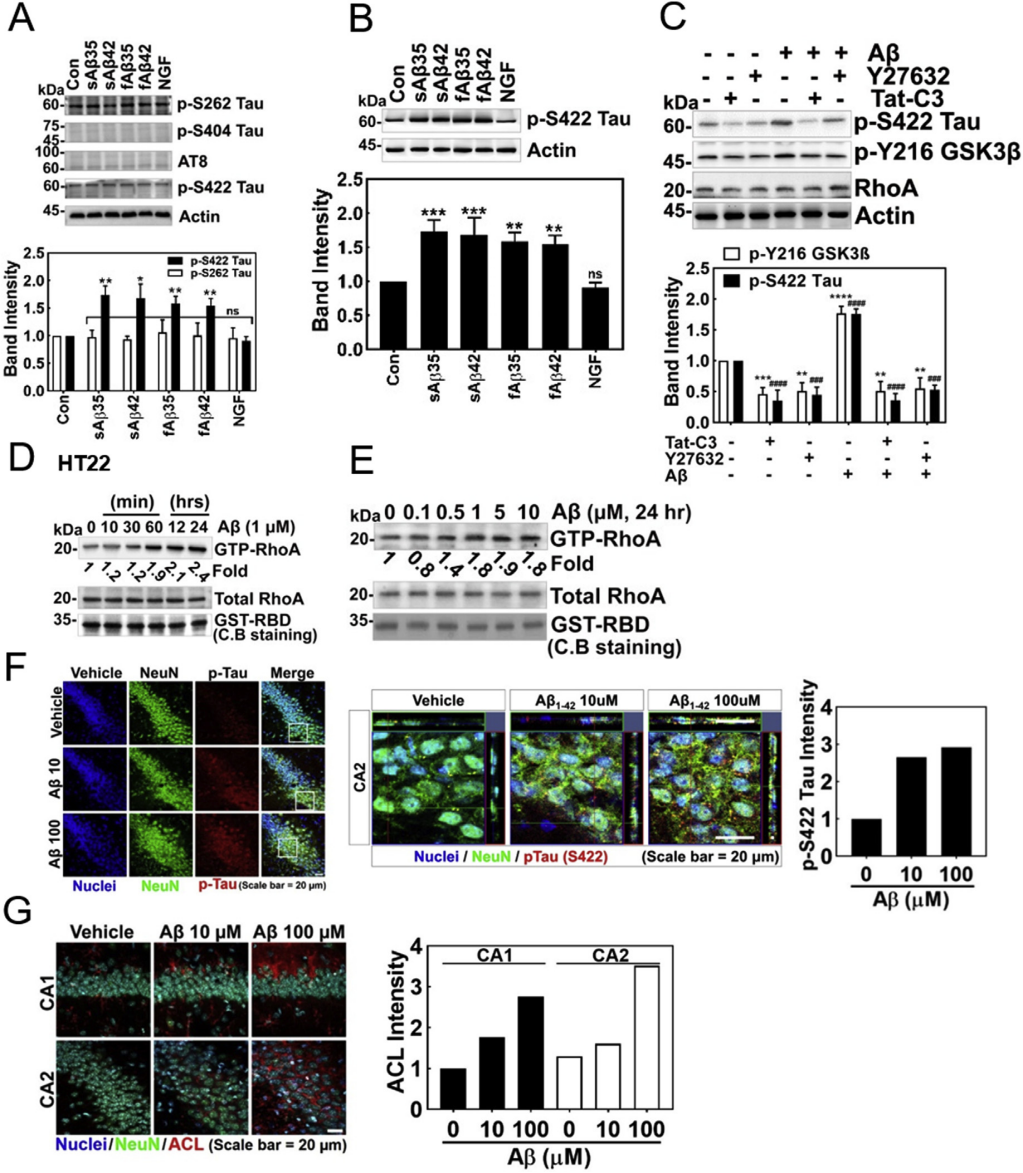
**Low concentrations of Aβ induce *p*-Ser422 tau through RhoA/ROCK/GSK3β signalling.** (**A** and **B**) Several types of p-Tau were detected with Western blot using specific antibodies after stimulating HT22 cells with soluble (s) or fibrillary (f) form of Aβ for short (Aβ25-35) or long (Aβ1-42) peptides (1 μM, 24 h). In one case, NGF (100 ng/ml) was treated for 72 h, and several types of p-Tau were assessed (one-way ANOVA, p*<0.05, **<0.01, ***<0.001). (**C**) Tat-C3 (Rho inhibitor, 1 μg/ml) and Y27532 (ROCK inhibitor, 10 μM) were pretreated for 1 h, and then fAβ (1 μM) was treated for 24 h to HT22 cells. P-Ser422 Tau, *p*-Tyr216 GSK-3β and RhoA were detected by Western blot (one-way ANOVA, p*<0.05, **<0.01, ***<0.001, ****<0.0001). **(D)** GTP-RhoA levels of HT22 cells were measured using a pulldown assay with GST-RBD after fAβ (1 μM) treatment for the given times, **(E)** or after various concentration of fAβ (1 μM) treatment for 24 h. **(F**–**G)** Immunofluorescence of a vehicle- and sAβ (10 μM or 100 μM)-injected CA1 and CA2 regions of C57BL/6 mice brains (4 months of age) was revealed with anti-NeuN (neuronal nucleus), *p*-Ser422 Tau, AT8 (*p*-Ser202/Thr205 Tau) and -p-Y42 Rho antibodies.

Next, we tried to elucidate the mechanism by which RhoA is engaged in Aβ-mediated AD progression. Since Aβ induces phosphorylation of Tau, we examined which phosphorylation site of Tau is responsive to Aβ stimulation in the HT22 mouse hippocampal cell line. We found that *p*-Ser422 of Tau, but not *p*-Ser202/Thr206, *p*-Ser262, and *p*-Ser404 was responsive to low concentration of Aβ stimulation (1 μM) in HT22 cells (Fig. 2A). We confirmed that low concentrations of Aβ (1 μM) including soluble and fibrillary Aβ25-35 and Aβ1-42 significantly increased Ser422 phosphorylation of Tau (*p*-Ser422 Tau) levels in HT22 cell, and quantified *p*-Ser422 Tau levels. In contrast, NGF reduced *p*-Ser422 Tau level (Fig. 2B). The phosphorylation of Ser422 Tau upon activation by Aβ1-42 was reduced by Tat-C3 (Rho inhibitor) and Y27532 (ROCK inhibitor) (Fig. 2C). GSK-3β was known to be a kinase to phosphorylate Tau protein, although what species of GSK-3β is involved in the process was not identified. Therefore, we explored whether the active form of GSK-3β, *p*-Tyr216 GSK-3β was induced by Aβ. Indeed, *p*-Tyr216 GSK-3β was increased by Aβ (1 μM), but was inhibited by Tat-C3 and Y27632 (Fig. 2C). These results suggest that Rho and ROCK regulate Ser422 phosphorylation of Tau through Tyr216 phosphorylated GSK-3β. Thereby, we assessed RhoA activation presented as RhoA-GTP levels upon Aβ treatment in HT22 cells; Aβ elevated RhoA-GTP levels in a time-dependent manner (Fig. 2D). We confirmed that RhoA-GTP was up-regulated with similar levels in the range between 1 and 10 μM of Aβ in HT-22 cells (Fig. 2E). An increase of *p*-Ser422 Tau was also observed in hippocampus of the Aβ-injected mouse brain (Fig. 2F). Sections of cells also revealed *p*-Ser422 Tau in Aβ-injected hippocampus region of mouse brain. Contrary to *in vitro* cell experiments, AT8 antibody also recognized *p*-Ser202/T205 Tau and revealed an observable response in the hippocampus of Aβ-injected mouse brain, suggesting that *p*-Ser202/T205 Tau may be likely induced in an indirect manner (Fig. 1, Fig. 2G).

### 3.3 Low concentrations of Aβ inhibit expression of ATP citrate lyase (ACL)

**Fig. 3:**
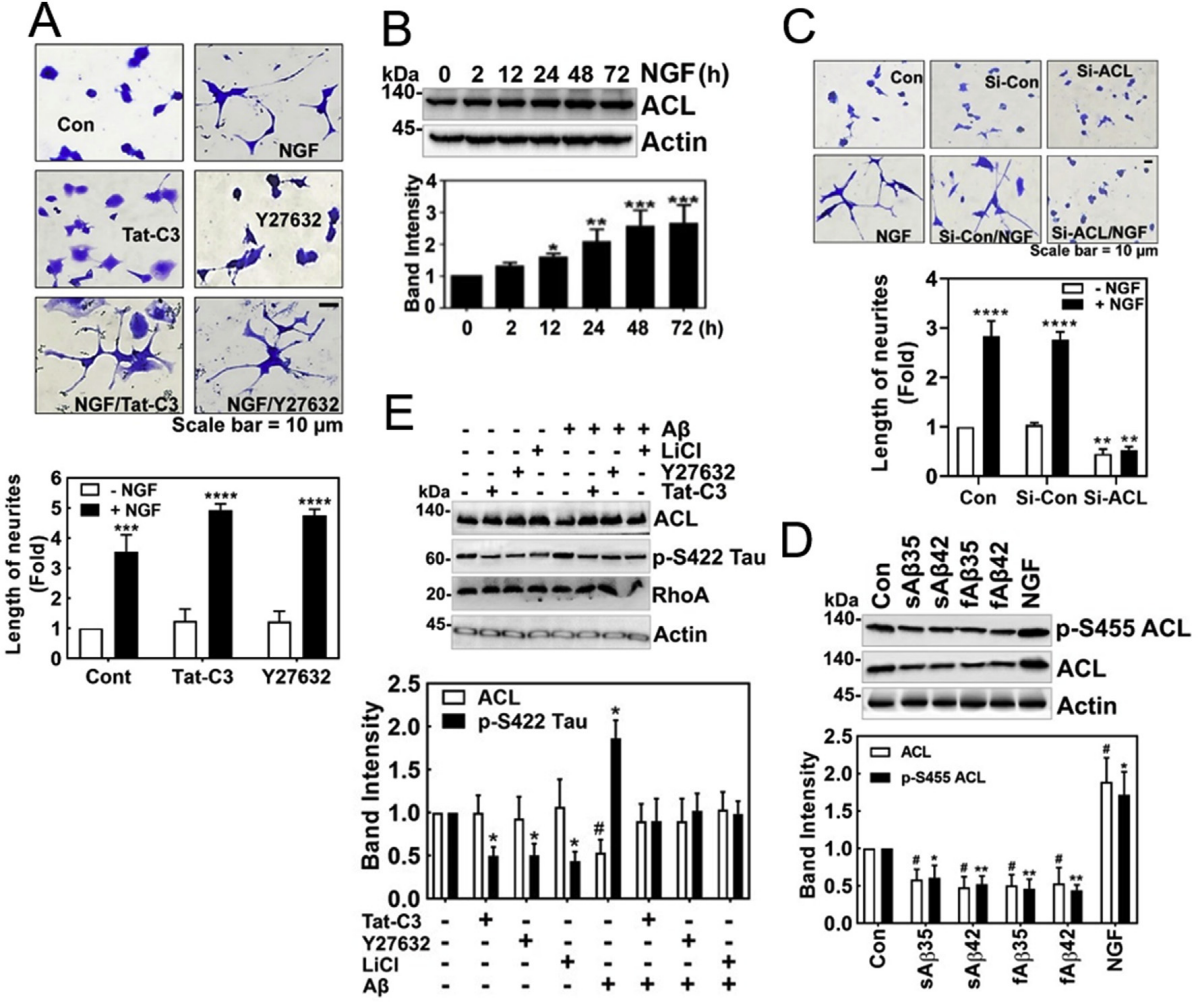
**Low concentration of Aβ inhibits the expression of ACL**. **(A)** PC12 cells were stimulated by NGF (100 ng/ml, 72 h) in the absence or presence of Tat-C3 (1 μg/ml) and Y27632 (10 μM). Neurite outgrowth from PC12 cells was revealed by Coomassie-Blue staining (two-way ANOVA, p***<0.001, ****<0.0001). (**B)** The level of ATP citrate lyase (ACL) was measured by western blotting in PC12 cells stimulated by NGF (100 ng/ml) from various periods (one-way ANOVA, p*< 0.05, **<0.01, ***<0.001). (**C)** The morphology of PC12 cell was shown after transfection si-ACL (50 nM) for 48 h followed by with or without NGF (100 ng/ml) for 72 h (two-way ANOVA, p**< 0.01, ****<0.0001). (**D)** HT22 cells were treated with soluble (s) or fibril (f) form of Aβ of short (Aβ25-35) and long (Aβ1-42) peptides (1 μM) for 24 h), and in a case, NGF (100 ng/ml) was treated for 72 h. ACL and *p*-Ser455 were assessed with western blotting (one-way ANOVA, *p < 0.05, **<0.01). (**E)** HT22 cells were pretreated with Tat-C3 (1 μg/ml), Y27632 (10 μM) and LiCl (10 μM) for 1 h, and then fAβ (1 μM) was treated for 24 h. The expression of ACL and p-S422 Tau protein was assessed by western blotting (one-way ANOVA, *p < 0.05). (For interpretation of the references to colour in this figure legend, the reader is referred to the Web version of this article.)

After we confirmed that Aβ impaired NGF-induced neurite outgrowth from PC12 cells (Fig. 1A), we further determined a regulatory mechanism of neurite outgrowth in the next series of experiments. Notably, inhibition of RhoA and ROCK by Tat-C3 and Y27632, respectively, enhanced neurite outgrowth (Fig. 3A) [[17], [18], [19]]. Neurite outgrowth should be associated with plasma membrane synthesis, which requires biosynthesis of fatty acids and lipids. There are three critical enzymes for fatty acid synthesis including ATP citrate lyase (ACL), acetyl-CoA carboxylase (ACC) synthesizing malonyl-CoA, a precursor of fatty acid synthesis, and fatty acid synthase (FS). Among them, ACL is the initiating enzyme to synthesize fatty acid in cytoplasm through cleavage of citrate into acetyl-CoA and oxaloacetate using ATP. We found that NGF induced the expression of ACL in a time-dependent manner (Fig. 3B), along with RhoA inactivation [17]. Indeed, si-ACL markedly interfered with the neurite outgrowth from PC12 cells upon NGF treatment, suggesting that ACL is pivotal for neurite outgrowth (Fig. 3C). It is notable that low concentrations of soluble and fibrillar Aβ interfered with the expression of ACL. Likewise, low concentrations of soluble and fibrillar Aβ impaired the levels of *p*-Ser455 ACL while NGF enhanced *p*-Ser455 ACL level (Fig. 3D). Furthermore, Tat-C3 (Rho inhibitor), Y27632 (ROCK inhibitor) and LiCl (GSK inhibitor) prevented an increase in *p*-Ser422 Tau levels, but increased ACL levels irrespective of Aβ, showing an inverses relationship between *p*-Ser422 Tau and ACL expression (Fig. 1, Fig. 2, Fig. 3E).

### 3.4 Levels of *p*-Ser422 tau and ACL show inversely reciprocal relationship depending on Aβ concentrations

**Fig. 4:**
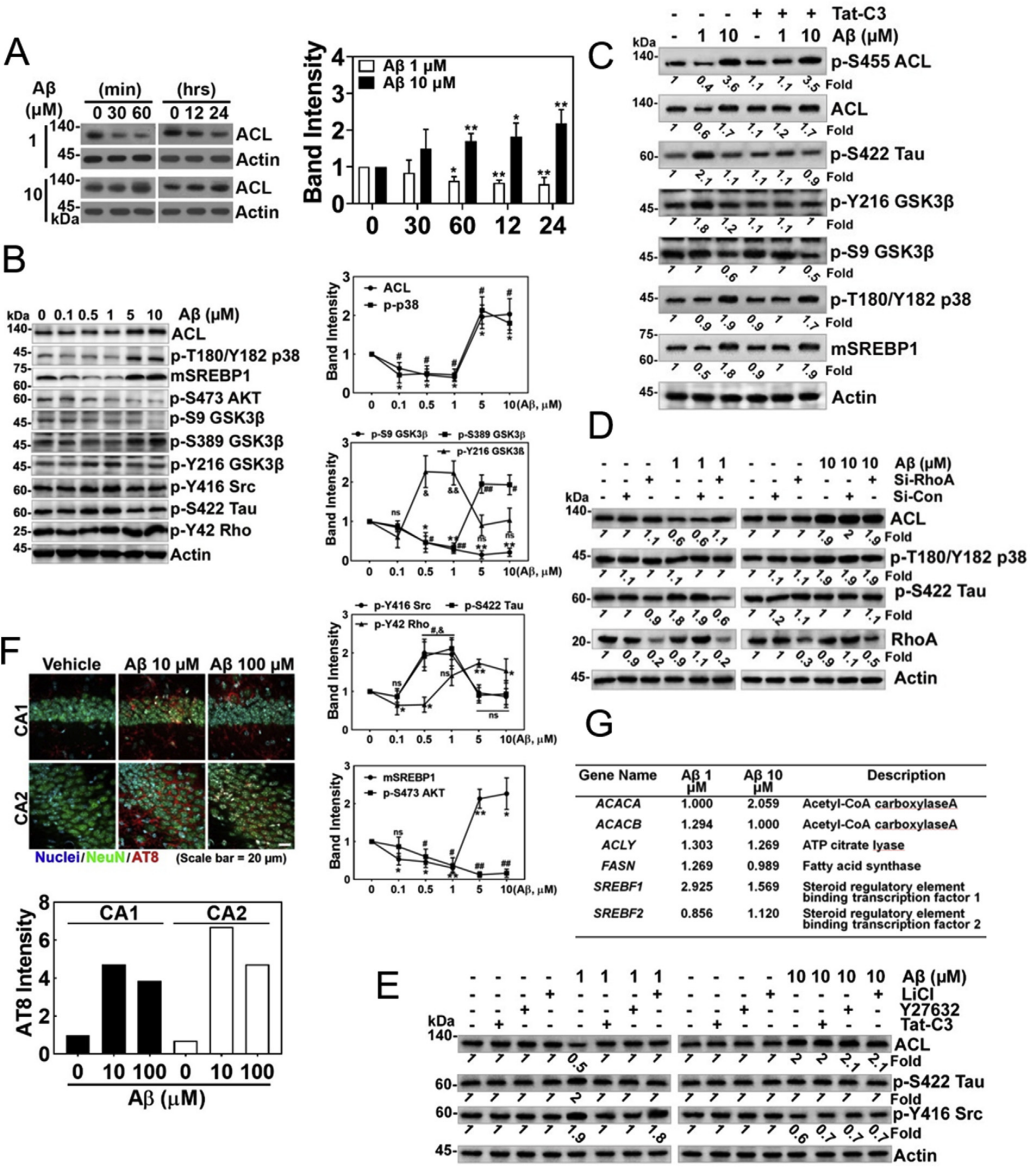
**Levels of *p*-Ser422 tau and ACL reveal inversely reciprocal relationship depending on Aβ concentrations. (A)** PC12 cells were treated with fAβ 1 μM or 10 μM for various period until 24 h, and ACL was assessed by western blotting (two-way ANOVA, p**< 0.05, **<0.01). (**B)** Various concentrations of Aβ (0–10 μM) were delivered to HT22 cells for 24 h, and several proteins likely related to the Aβ signalling pathway were assessed by western blotting with each specific antibody (one-way ANOVA, *p < 0.05, **<0.01). **(C)** HT22 cells were pretreated with Tat-C3 (1 μg/ml) for 1 h, and then treated with fAβ (1 μM or 10 μM) for 24 h, and then several proteins related to the several proteins likely related to the Aβ signalling pathway were assessed by western blotting with each specific antibody. (**D)** HT22 cells were transfected with 50 nM si-RhoA or si-control RNA for 48 h, and then stimulated with fAβ of 1 μM or 10 μM for 24 h, and then ACL, p-T180/Y182 p38 and *p*-Ser422 Tau were determined with western blotting. **(E)** HT22 cells were pretreated with Tat-C3 (1 μg/ml), Y27632 (10 μM), or LiCl (10 μM) for 1 h, then were treated with fAβ (1 μM or 10 μM) for 24 h, and ACL, *p*-Ser422 Tau, *p*-Tyr416 Src were determined with western blotting. **(F)** Hippocampal regions of C57BL/6 mice brains were injected with sAβ (10 μM or 100 μM) and CA1 and CA2 regions were stained with DAPI for nucleus, NeuN antibody for neuronal nucleus, and ACL antibody. (**G**) HT22 cells were stimulated with 1 or 10 μM Aβ for 24 h, and then change of mRNA expression was analysed by transcriptome. Genes related to lipid metabolism were revealed.

In the following experiments, we examined the effects of low and high concentrations of Aβ on ACL expression. We found that the two Aβ concentrations show inverse effects; low concentrations of Aβ (1 μM) reduced ACL level, while high concentrations of Aβ (10 μM) increased ACL levels in PC12 cells (Fig. 4A). Furthermore, low concentrations of Aβ (0.1–1 μM) also reduced the levels of mature active fragment of sterol regulatory element-binding protein 1 (mSREBP1) and *p*-Ser389 GSK-3β (inactive) as well as ACL, while increased *p*-Tyr216 GSK-3β (active), *p*-Tyr416 Src (active), *p*-Ser422 Tau in HT22 cells (Fig. 4B). In contrast, high concentrations of Aβ (5–10 μM) increased levels of *p*-Thr180/Tyr182 p38 (active), mSREBP1, and *p*-Ser389 GSK-3β (inactive), while attenuated *p*-Tyr216 GSK-3β (active), *p*-Tyr416 Src (active), and *p*-Ser422 Tau as well as ACL in HT22 cells (Fig. 4B). As *p*-Tyr42 RhoA was reported to be active [13], we tried to clarify whether the signalling pathway is governed by *p*-Tyr42 Rho GTPase upon Aβ stimulation. P-Tyr42 Rho was increased by high concentration of Aβ (5–10 μM), while low concentration of Aβ (0.1–0.5 μM) reduced *p*-Tyr42 Rho levels (Fig. 4B). Hereby, we examined the Tat-C3 effect on the downstream components; P-Ser422 Tau and *p*-Tyr216 GSK-3β were down-regulated, but *p*-Ser455 ACL (active), ACL, *p*-Ser9 GSK-3β (inactive), *p*-Thr180/Tyr182 p38 (active) and mSREBP1 were not influenced by Tat-C3 (Fig. 4C). Consistent with this result, si-RhoA impaired *p*-Ser422 Tau and restored ACL levels upon low concentrations of Aβ, but upon high concentrations of Aβ, si-RhoA did not influence ACL, p-T180/Y182 p38 and *p*-Ser422 Tau levels (Fig. 4D). The results suggest that there are at least two signalling pathways depending on Aβ concentrations. In addition, low concentration of Aβ (1 μM)-induced *p*-Tyr416 Src was inhibited by Tat-C3 and Y27632, suggesting that RhoA and ROCK regulate *p*-Tyr416 Src (active) upon low concentrations of Aβ, but LiCl (GSK-3β inhibitor) did not impair *p*-Tyr416 Src. However, Tat-C3, Y27632 and LiCl did not effect on the levels of ACL, *p*-Ser422 Tau, and *p*-Tyr416 upon high concentration of Aβ (10 μM) (Fig. 4E). Injection of high concentration of Aβ (100 μM) to the hippocampal region of mouse brain induced more ACL expression in CA1 and CA2 regions of hippocampus of rat brain (Fig. 4F). Transcriptome analysis was performed in HT-22 cells, which were stimulated by 1 or 10 μM Aβ. Different concentrations of Aβ revealed different genes expression (data not shown). Among the expressed genes, we analysed transcription levels of genes related to synthesis of for fatty acids and lipids. In particular, expression of the *SREBF1* gene encoding SREBP1 was increased by both 1 and 10 μM Aβ and expression of the *ACACA* gene encoding acetyl-coA carboxylase A were increased by 10 μM Aβ (Fig. 4G).

### 3.5 High concentration of Aβ induces superoxide and induces cellular senescence

Aβ induced superoxide generation in time- and concentration-dependent manners in HT22 cells (sFig. 2A). Aβ also enhanced superoxide level in PC12 cells, but NGF abrogated the superoxide levels (sFig. 2B). Moreover, Aβ also induced superoxide production in LN18 cells (astrocyte cell line) as well as BV2 cells (microglial cell line). In particular, BV2 cells upon Aβ produced much more superoxide than either HT-22 or LN18 cells, compared to Aβ-untreated each control cell (sFig. 2C and 2D). BV2 microglial cell line is likely to have similar features to macrophage, which contains a high NADPH oxidase level. High concentration of Aβ produced superoxide in early period and remained the superoxide levels until 24 h in HT22 cells (sFig. 2E). Meanwhile, Tat-C3 (Rho inhibitor) and NSC23766 (Rac inhibitor) slightly reduced superoxide production in response to 10 μM Aβ for 24 h (sFig. 2E and sFig. 2F), suggesting that Rho and Rac1 are partially involved in superoxide production in response to Aβ in HT22 cells. Then, we tested the effects of inhibitors of NADPH oxidase or mitochondria on superoxide upon Aβ treatment. Either apocynin and DPI (inhibitors of NADPH oxidase) or Mito-TEMPO (an inhibitor of superoxide production in mitochondria) markedly attenuated superoxide generation, suggesting that both NADPH oxidase and mitochondria are essential for superoxide production upon Aβ (sFig. 2G).

**Fig. 5:**
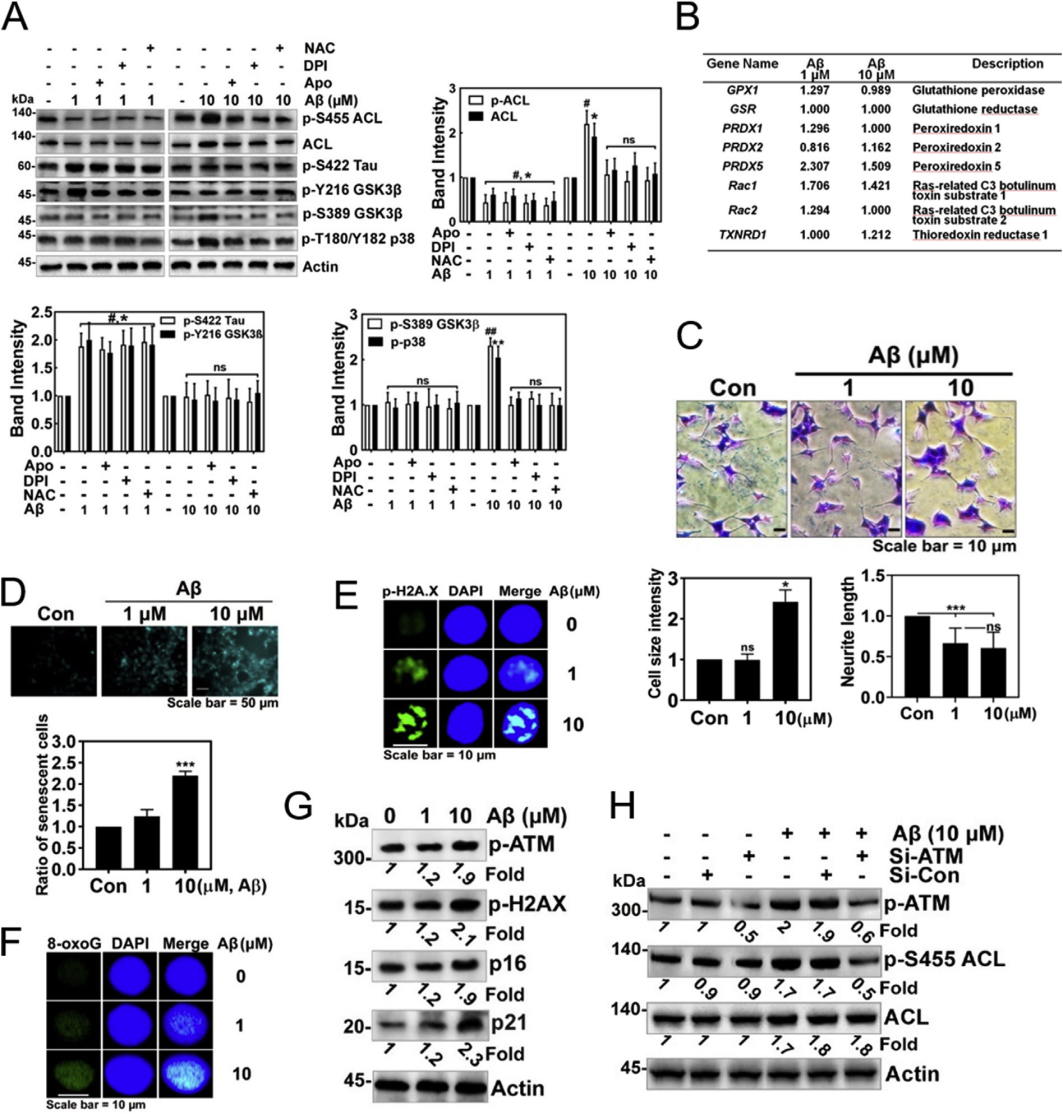
**High concentrations of Aβ induces superoxide production and cellular senescence.** (**A)** HT22 cells were pre-treated with NAC (10 mM), DPI (10 μM), or apocynin (1 μM) for 1 h, then treated with fAβ (1 or 10 μM) for 24 h, and several proteins likely related to Aβ signalling pathways were assessed by Western blotting with each specific antibody (two-way ANOVA, p*<0.05, **<0.01, ***<0.001). **(B)** HT22 cells were stimulated with 1 or 10 μM Aβ for 24 h, and then change of mRNA expression was analysed by transcriptome. Genes related to redox regulation were revealed. **(C)** HT22 cells were treated fAβ (1 or 10 μM) for 24 h and stained by Coomassie-Blue and the cell size and neurite outgrowth levels were determined (one-way ANOVA, p*<0.05, **<0.01, ***<0.001). **(D)** HT22 cells were treated with fAβ (1 or 10 μM) for 24 h, then stained using the senescence β-galactosidase staining kit, and the relative extent of senescent cells was determined (one-way ANOVA, p***<0.001). (**E, F**) HT22 cells were treated with fAβ (1 or 10 μM) for 24 h, fixed and immunostained with anti-*p*-Ser139 H2A.X **(E)** or -8-oxoG antibody (**F**), and nucleus was stained with DAPI. Secondary AlexaFluor 488-conjugated antibody was used for green fluorescence and DAPI was used for visualization of nuclei. **(G)** HT22 cells were treated with fAβ (1 or 10 μM) for 24 h, the change of proteins related to DNA damage and cellular senescence were determined with Western blotting. **(H)** HT22 cells were transfected with 50 nM si-ATM or si-control RNA, then treated with fAβ 10 μM for 24 h, and the expression of ACL protein levels were determined with Western blotting. (For interpretation of the references to colour in this figure legend, the reader is referred to the Web version of this article.)

**Fig. 6:**
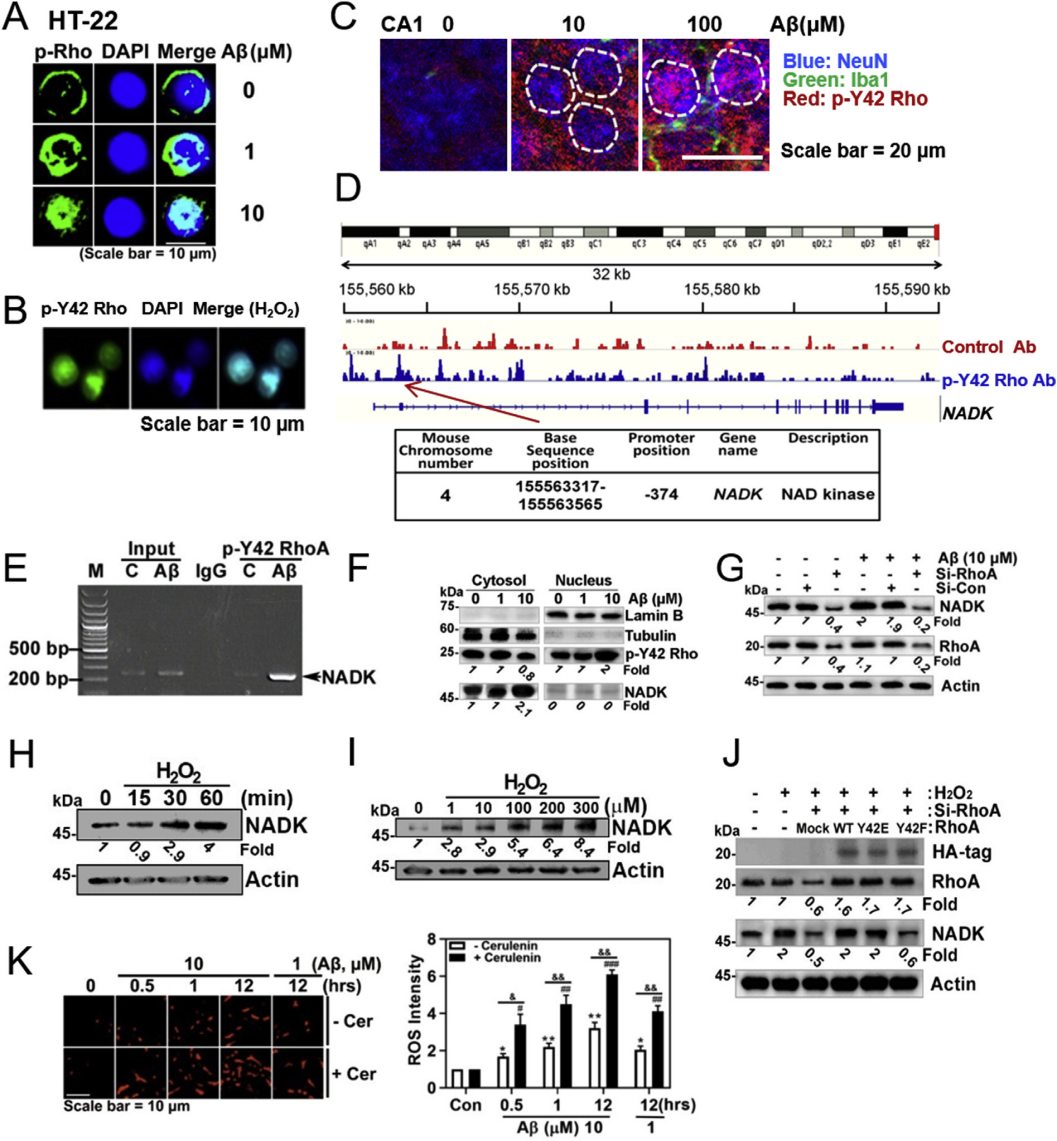
**Nuclear *p*-Tyr42 RhoA regulates NADK expression. (A)** HT22 cells were treated with fAβ (1 or 10 μM) for 24 h. The localization of *p*-Tyr42 Rho nucleus was assessed using an immunofluorescence method with the *p*-Tyr42 Rho antibody and a secondary AlexaFluor 488-conjugated antibody (green) and the nucleus was stained with DAPI. **(B)** HEK293 cells were treated with H_2_O_2_ (100 μM) for 1 h and then *p*-Tyr42 Rho localization was detected. **(C)** CA1 region of C57BL/6 mice brain shown in Fig. 1H (noted as square box) was magnified to see nuclear localization of *p*-Tyr42 Rho. sAβ (10 μM or 100 μM) was injected to CA1 region of C57BL/6 mice brains, and anti-NeuN (neuronal nucleus marker), -Iba-1 (microglial marker), and -p-Y42 Rho antibodies were used for immunofluorescence image. **(D)** Chromatin immunoprecipitation (ChIP) with a *p*-Tyr42 Rho antibody was performed in 4T1cells and then DNA sequencing was conducted. One of the target promoter base sequences of specific genes bound with *p*-Tyr42 Rho was revealed to be that of *NADK*. (**E)** HT22 cells treated with fAβ (1 or 10 μM) for 24 h were lysed and fractionated to cytosolic and nuclear fractions. P-Tyr42 Rho and NADK were detected with western blotting. Tubulin and lamin B were used for markers of cytosolic and nuclear fractions. **(F)** HT22 cells were transfected with 50 nM si-RhoA or control si-RNA, and stimulated with fAβ 10 μM for 24 h. RhoA and NADK protein levels were detected with western blotting. **(G)** HT22 cells were stimulated with 10 μM Aβ for 24 h and ChIP PCR was performed with NADK DNA primers. **(H, I)** HEK293 cells were stimulated with H_2_O_2_ (100 μM) for various periods **(H)**, and stimulated with various concentrations of H_2_O_2_, (0–300 μM) for 2 h **(I)**. **(J)** HEK293 cells were transfected with 50 nM si-RhoA and reconstituted with mock, RhoA WT, Y42E and Y42F plasmid DNA, and NADK expression was measured with western blotting. **(K)** HT22 cells were pre-treated with cerulenin (10 μM) for 1 h, then treated with fAβ (1 μM or 10 μM), and superoxide level was measured (two-way ANOVA, p*<0.05, **<0.01, ***<0.001). (For interpretation of the references to colour in this figure legend, the reader is referred to the Web version of this article.)

Then, we explored whether superoxide produced by Aβ stimulation is involved in the regulation of Ser422 phosphorylation of Tau protein in HT22 cells; low concentration of Aβ (1 μM)-mediated up-regulated *p*-Ser422 Tau was not altered by apocynin and DPI (possible inhibitors of NADPH oxidase) and NAC (ROS scavenger). However, high concentration of Aβ (10 μM)-maintained *p*-Ser455 ACL, ACL, *p*-Ser389 GSK-3β (inactive) and p-T180/Y182 p38 (inactive) were reduced by apocynin, DPI and NAC (Fig. 5A). Transcriptome analysis was performed in HT-22 cells, which were stimulated by 1 or 10 μM Aβ. Transcription levels of several genes of anti-oxidant enzymes or proteins were not significantly changed, but peroxiredoxin 5 (an antioxidant protein) and Rac1 (an activator of NADPH oxidase) were increased by both 1 and 10 μM of Aβ (Fig. 5B). Notably, high concentration of Aβ (10 μM) ensured an increase in the size of HT22 cell bodies, but reduced the length of processes (Fig. 5C). In addition, Aβ (10 μM) induced the expression of senescent β-galactosidase, which is a marker of cellular senescence (Fig. 5D). Meanwhile, p-H2AX (referred to also as γH2AX), an indicator of DNA impairment was enhanced by 10 μM Aβ (Fig. 5E and sFig. 2H). In addition, 8-oxoguanine, a marker of DNA oxidation was also increased by 10 μM Aβ (Fig. 5F and sFig. 2I). In addition to p-H2AX/γ-H2AX, other markers for cellular senescence, p16^INK4a^ (cyclin-dependent kinase inhibitor 2A, multiple tumor suppressor 1) and p21^CIP1/WAF1^ CDK inhibitors (CDKIs) were also markedly up-regulated by 10 μM Aβ (Fig. 5G). Markedly, *p*-Ser1981 ataxia telangiectasia mutated (ATM) serine/threonine kinase was also increased, suggesting that DNA damage by superoxide activates ATM. Furthermore, si-ATM reduced *p*-Ser455 ACL, which was stimulated by high concentration of Aβ (Fig. 5, Fig. 6H). This result suggests that *p*-Ser1981 ATM activated by Aβ is a main kinase to phosphorylate Ser455 with activating ACL.

### 3.6 Nuclear *p*-Tyr42 RhoA regulates NADK expression

Surprisingly, we found that high concentrations rather than low concentrations of Aβ facilitated nuclear translocation of *p*-Tyr42 Rho in HT-22 cells (Fig. 6A and sFig. 3A). Hydrogen peroxide also caused nuclear localization of *p*-Tyr42 Rho (Fig. 6B), suggesting that nuclear translocation of *p*-Tyr42 Rho induced by Aβ can be attributed to ROS. Injecting high concentrations of Aβ (100 μM) rather than 10 μM induced a greater degree of nuclear translocation of *p*-Tyr42 Rho in neurons of CA1 region of hippocampus in rat brain (Fig. 6C). When we performed chromatin immunoprecipitation (ChIP) sequencing by using *p*-Tyr42 Rho antibody in response to ROS, we found that one of the target genes of *p*-Tyr42 Rho was *NADK* gene (NAD kinase: NADK) (Fig. 6D). ChIP PCR from the nuclear fraction of HT22 cells with *p*-Tyr42 Rho antibody and *NADK* promoter primers demonstrated that *p*-Tyr42 Rho binds to the promoter of *NADK* upon Aβ stimulation (Fig. 6E). We verified again that *p*-Tyr42 Rho exits in the nucleus upon Aβ treatment (10 μM) with western blotting. Remarkably, we demonstrated that Aβ (10 μM) induced an increase in the expression of NAD kinase (NADK) (Fig. 6F), which catalyses conversion of NAD to NADP. We demonstrated again that si-RhoA abolished NADK expression occurs in response to high concentrations of Aβ (Fig. 6G). Moreover, we provided evidence that ROS such as hydrogen peroxide induced NADK expression in a time- and concentration-dependent manners (Fig. 6H and I). The result suggests that NADK expression by Aβ may be attributed to ROS. Therein, we examined the *p*-Tyr42 residue of RhoA effect on NADK expression upon hydrogen peroxide stimulation; si-RhoA attenuated NADK expression while reconstitution of WT and RhoA Y42E (phosphor-mimic) restored NADK expression. However, reconstituted RhoA Y42F (dephospho-mimic) was not able to induce NADK expression, suggesting that *p*-Tyr42 residue of RhoA is crucial for NADK expression (Fig. 6J).

Since not only NADPH oxidase but also FS requires NADPH as a hydrogen donor, we examined the level of superoxide production by inhibiting FS activity; cerulein, FS inhibitor enhanced superoxide production upon Aβ (Fig. 6K and sFig. 3B), suggesting that more NADPH is favourably utilized by NADPH oxidase in the presence of FS inhibitor, cerulein. Accordingly, cerulein caused slightly more cell death upon 24 h exposure of Aβ (sFig. 3C).

### 3.7 AD patient specimens show similar features of Aβ signalling in HT22 cells

**Fig. 7:**
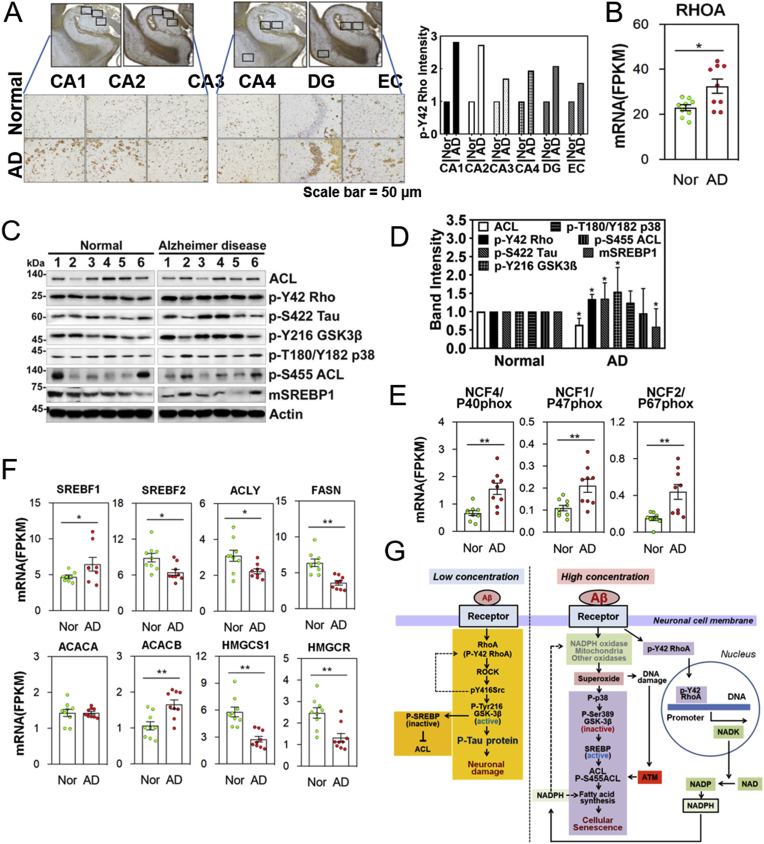
**AD patient specimens also show similar features of the Aβ signalling pathway in HT22 cells. (A)** Immunohistochemistry for p-Y42 Rho in the CA1, CA2, CA3, CA4, DG, and EC regions of the hippocampus of control normal persons and AD patients (Scale bar = 50 μm). **(B)** RNA expression of RhoA in temporal cortex of normal and AD brain was analysed. (**C,D**) Several proteins likely related to Aβ signalling pathway were analysed with western blotting in brain tissues of normal controls and AD patients **(C)** and relative intensity of protein bands was quantified. (unaired, two-tailed *t*-test, p* < 0.05). **(D)**. **(E,F)** Expression of mRNA levels in normal and AD patients were analysed by transcriptome analysis: NADPH oxidase components **(E)** and enzymes for fatty acid and lipid synthesis. Data are means of 6–9 independent samples ± SE (unaired, two-tailed *t*-test, p**<0.01) **(E,F)**. **(G)** Schematic illustration showing two Aβ-dependent concentration signalling pathways: low concentrations of Aβ (1 μM) can activate RhoA/ROCK/p-Tyr216 GSK-3β, causing *p*-Ser422 tau and neuronal damage. On the other hand, when *p*-Tyr216 GSK-3β (active form) is phosphorylated and *p*-SREBP1 is ubiquitinated and degraded, a transcription factor to induce enzymes for lipid synthesis including ACL, a decrease of ACL expression is seen. High concentrations of Aβ (10 μM) produces enhanced levels of ROS and phosphorylates p38 MAPK (T180/Y182), which can inactivate GSK-3β by phosphorylating Ser389 and, in turn, activates SREBP1, leading to an increase of ACL level as well as fatty acid synthesis and cellular senescence. High ROS cause DNA damage which activates ATM by phosphorylating Ser1981 then phosphorylates ACL Ser455. High concentration of Aβ (10 μM) also facilitates nuclear translocation of *p*-Tyr42 Rho, associating with the promoter region of NADK gene, thereby likely resulting in high level of NADP and NADPH. Finally, NADPH can be a hydrogen donor for FS activity as a defence mechanism rather than NADPH oxidase activity that induces highly ROS, thereby leading to cell death.

**Fig. S1:**
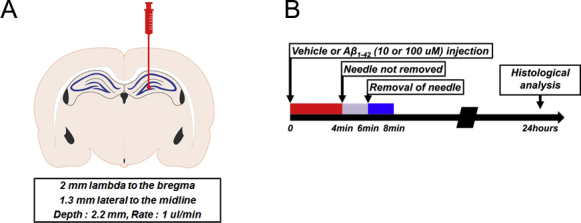
**Effect of Aβ on hippocampal neuron of mouse brain**. **(A, B)** Method of microinjection of Aβ to hippocampal neuron of mouse brain was described. Aβ (10 or 100 μM) was injected to hippocampal region of C57BL/6 mouse brain (4 months of age).

**Fig. S2:**
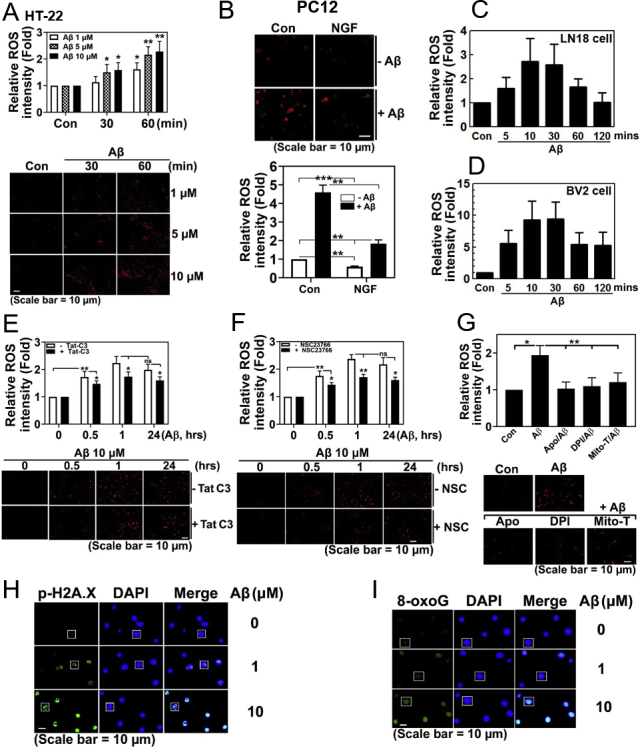
Aβ induces superoxide production. Dihydroethidium was used to directly measure superoxide levels in live cells (panels A–F). (A) Various concentrations of fAβ were treated to HT22 cells for various periods and superoxide levels were determined (two-way ANOVA, p*< 0.05, **< 0.01, ***< 0.001). (B) PC12 cells were treated with Aβ (1 μM) for 24 h in the absence or presence of NGF (100 ng/ml), and superoxide levels were determined (two-way ANOVA, p*< 0.05, **< 0.01, ***< 0.001). (C) LN18 cells (astrocyte cell line) and (D) BV2 cells (microglial cell line) were treated with Aβ (1 μM) and superoxide levels were determined. (E) HT22 cells were pre-treated with Tat-C3 (1 μg/ml) or (F) with NSC23766 (50 μM) for 1 h, then treated with fAβ (10 μM) for 24 h, and superoxide levels were determined (two-way ANOVA, p*< 0.05, **< 0.01, ***< 0.001). (G) HT22 cells were pre-treated with apocynin (1 μM), DPI (10 μM), or Mito-TEMPOL (100 μM) for 1 h, then treated with fAβ (10 μM) for 24 h, and then superoxide levels were determined (one-way ANOVA, p*< 0.05, **< 0.01, ***< 0.001). (H,I) DNA damage by high concentration of Aβ (10 μM) was shown: (H) Uncropped image of cells shown in Fig. 5E; (I) Uncropped image of Fig. 5F.

**Fig. S3:**
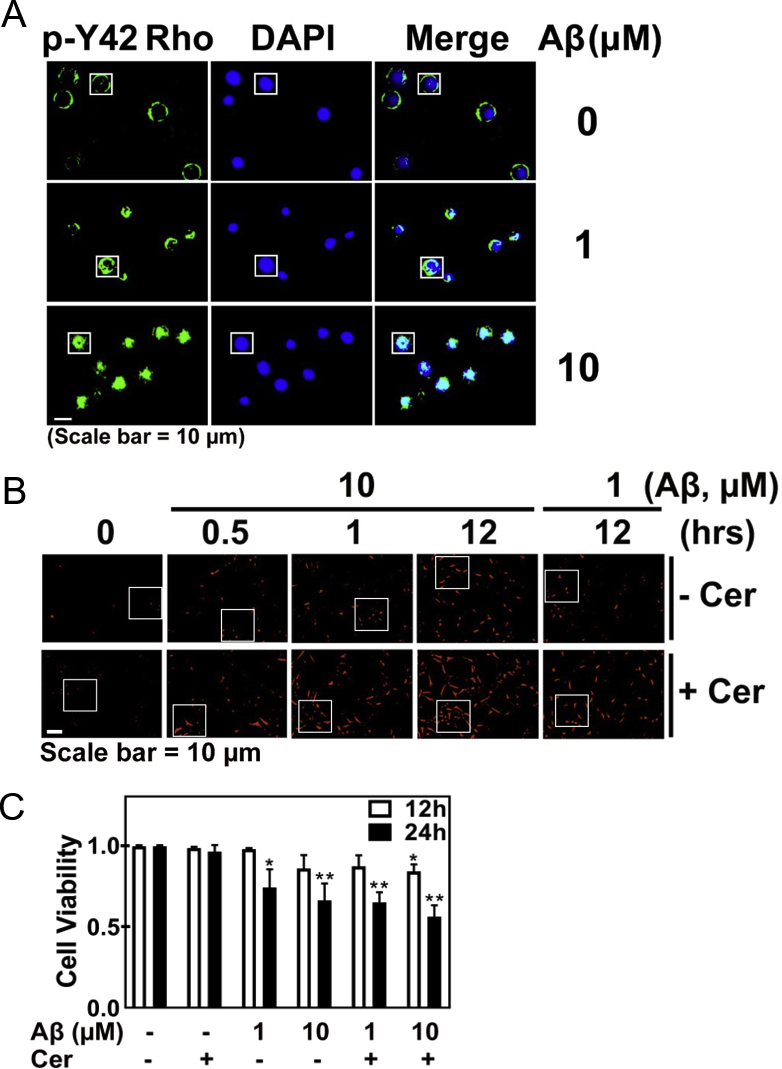
Nuclear localization of *p*-Tyr42 RhoA. (A) Uncropped image of Fig. 6A. (B) Uncropped image of Fig. 6K. (C) HT22 cells were pretreated with or without cerulenin (10 μM) for 1 h, and treated with of Aβ (1 or 10 μM) for 12 or 24 h. Cell viability was determined by MTT assay (two-way ANOVA, p*< 0.05, **< 0.01, ***< 0.001).

**Fig. S4:**
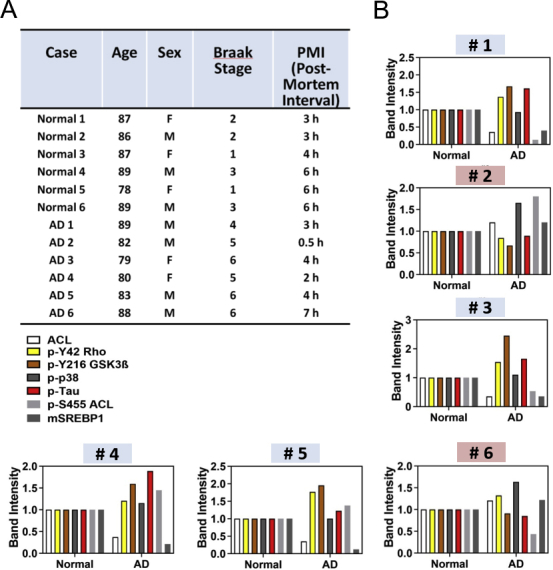
Assessment of brain tissues of AD patients. (A) Table of Information on postmortem brain tissue samples from temporal cortex of normal subjects and AD patients. (B) Quantification of Fig. 7B.

In addition, *p*-Tyr42 Rho was also increased in CA1, CA2, DG (dentate gyrus), and EC (entorhinal cortex) regions of human AD patients (Fig. 7A). Indeed, RhoA expression was significantly enhanced in AD patient brain (Fig. 7B). Then, we analysed six samples of human AD patients and found that p-S422 Tau and *p*-Tyr216 GSK-3β were correlated each other, whereas *p*-Thr180/Tyr182 p38 MAPK were inversely and reciprocally correlated with *p*-Ser422 and pTyr216 GSK-3β; Patients 2 and 6 showed the features of high concentration Aβ effect on HT22 cells while patients 1, 3 and 4 showed the features of low concentration Aβ effect on HT22 cells; patient 5 did not follow the typical classification (Fig. 7C, sFig. 4A and sFig. 4B). However, ACL and mSREBP were statistically reduced, but *p*-Tyr42 Rho, *p*-Ser422 Tau, *p*-Tyr216 GSK-3β (active) were increased from total six specimens (Fig. 1, Fig. 2, Fig. 3, Fig. 4, Fig. 5, Fig. 6, Fig. 7, Fig. S1, Fig. S2, Fig. S3, Fig. S4D).

Next, we analysed mRNA levels in human AD patients. Intriguingly, expression of p40phox, p47phox and p67phox was significantly enhanced in AD brain, suggesting that superoxide could be favourably produced in AD (Fig. 7E). Expression of *SREBF1* (SREBP1 protein, which is involved in fatty acid synthesis) and *ACACB* (acetyl-CoA carboxylase-β, which is thought to inhibit fatty acid oxidation) were increased, while expression of *SREBF2* (SREBP2 protein, which is involved in cholesterol synthesis), *ACLY* (ATP citrate lyase), *FASN* (fatty acid synthase), *HMGCS1* (HMG-CoA synthetase), and *HMGCR* (HMG-CoA reductase) were significantly decreased in AD patients. *ACACA* (acetyl-CoA carboxylase-α, which is enriched in lipogenic tissue) was not different from the control (Fig. 7F). Expressions of many genes related to regulation of redox states were analysed, but it was vulnerable to interpret the changes of the genes expression (data not shown). Taken together, we propose there are at least two signalling pathways of Aβ; low and high concentrations of Aβ up-regulate p-Tau and ACL, respectively (Fig. 7G).

## 4 Discussion

### 4.1 Regulation of *p*-Ser422 tau protein levels through RhoA, ROCK and GSK-3β activity upon low concentration of Aβ

It has been well-established that RhoA activation generally interferes with neurite initiation, neuronal differentiation, and furthermore induced neurite retraction [11]. One of the mechanisms by which RhoA interferes with neurite outgrowth is that ROCK activated by RhoA phosphorylates collapsin response mediator protein-2 (CRMP-2), leading to its inactivation and microtubule instability, because dephosphorylated CRMP-2 stabilizes microtubule supporting neurite outgrowth [20]. Another mechanism is that RhoA activates myosin, resulting in F-actin/myosin interaction and cell contraction [21]. In addition, active RhoA impairs dendritic arborisation and dendritic spine formation, leading to synapse aberration [11]. Furthermore, abnormal activation of Rho GTPase may increase toxic Aβ [12,22] and RhoA is in turn activated in AD, leading to neuronal loss [23]. However, *p*-Tyr42 RhoA involvement in brain degenerative diseases has never been studied.

In this study, we found that *p*-Tyr42 Rho was increased in neurons of both an AD model mouse and human AD patients. In particular, *p*-Tyr42 Rho was enriched in neurons, particularly the cell body, distal dendrites or axon terminals, which form synapses on each other. Furthermore, *p*-Tyr42 Rho enriched in the neuronal cell body, SLM, and SR regions may lead to a possible aberration of synaptic function due to impairment of dendritic spines (Fig. 1F, G and 1H).

Phosphorylation of the Tau protein is critical for tauopathies and numerous phosphorylation sites on the Tau protein have been reported. Among them, *p*-Ser422 Tau is also implicated in AD pathogenesis; *p*-Ser422 Tau was significantly elevated in AD patients [24,25]. In addition, vaccination with *p*-Ser422 Tau peptide against *p*-Ser422 Tau in mouse decreased the insoluble portion of Tau protein and improved cognitive deficits promoted by Tau pathology in a well-defined Tau transgenic model [26]. However, the mechanism, by which Ser422 of Tau protein is phosphorylated, has not been well established.

In this study, we found that active *p*-Tyr42 Rho induces *p*-Ser422 Tau (Fig. 2). Moreover, we delineated that *p*-Tyr42 Rho, ROCK and *p*-Tyr216 GSK-3β are involved in regulation of *p*-Ser422 Tau level in response to low concentration of Aβ (1 μM) in Aβ-mediated AD progression. Herein, we propose a new signalling pathway sequence as follows: low concentrations of Aβ → *p*-Tyr42 RhoA → ROCK → Src → *p*-Tyr216 GSK-3β → p-Tau. Although it was not clearly revealed how ROCK Ser/Thr kinase activates Src (Fig. 4E), there is an example that other effector proteins of RhoA, such as mDia, activates Src [27]. Indeed, Src was reported to induce Tyr216 phosphorylation of GSK-3β in PC3, prostate cancer cells [28]. In addition, Src also can phosphorylate the Tyr42 residue of RhoA [13]. Apart from Tyr416 phosphorylation, Src can also be activated by oxidation events, such as an intramolecular disulfide bond between Cys245 and Cys487. However, oxidation forming intermolecular disulfide bond between two Cys277 residues of Src by likely high ROS results in its inactivation. Hereby, we surmise that different concentrations of Aβ resulting in different superoxide concentrations drove the opposing Src activities (Fig. 4B and E). Although low concentrations of Aβ activated GSK-3β *via* Tyr216 phosphorylation, leading to Ser422 phosphorylation of Tau, high concentrations of Aβ inhibited GSK-3β activity *via* Ser389 phosphorylation (Fig. 4B). Meanwhile, ROS were reported to activate Akt with *p*-Thr308 and *p*-Ser473 [29], but we observed *p*-Ser473 Akt was reduced along with *p*-Ser9 GSK-3β (Fig. 4B) by 10 μM Aβ [30]. Increased GSK-3β activity by 5 μM Aβ (high concentration) may induce phosphorylation at another site of Tau instead of Ser422 of Tau [30,31].

Indeed, *p*-Ser422 Tau was significantly increased in AD patients (Fig. 7A). Because high concentrations of Aβ, however, induced a decrease of *p*-Ser422 Tau (Fig. 4B, C and 4E), it is likely that severely advanced AD patients along with high Aβ accumulation exhibit a relatively low level of *p*-Ser422 Tau along with an increase of *p*-Ser455 ACL in patients number 2 and 6 (Fig. 7C and sFig. 5C).

### 4.2 Regulation of ACL expression by high concentrations of Aβ

We found that high concentrations of Aβ produced high levels of superoxide (sFig. 3A). As RhoA and Rac1 are involved in the regulation of NADPH oxidase during phagocytosis [[32], [33], [34]], Rho and Rac were partially engaged in Aβ-mediated superoxide generation (sFig. 3E and sFig. 3F). In addition, we observed possible inhibitors of NADPH oxidase including apocynin and DPI or an inhibitor of mitochondrial ROS such as Mito-TEMPO abolished ROS production to basal levels even in the presence of high concentrations of Aβ (sFig. 3G); blockade of only one source to produce ROS between NADPH oxidase and mitochondria suppressed superoxide production. We consider this is to be attributed to a cross-talk between mitochondria and NADPH oxidase. In fact, it has been known that mitochondria and NADPH oxidase stimulate each other, thereby leading to a feed-forward vicious cycle [[35], [36], [37]]. Here, involvement of NADPH oxidase in superoxide production upon Aβ is not confirmative because it is not clear that apocynin and DPI are specific inhibitors of NADPH oxidase [38,39]. However, NADPH oxidase indeed exits in hippocampal neuron and plays a critical role to regulate neuronal polarity [40].

Superoxide produced by high concentrations of Aβ mainly induced an increase of p-T180/Y182 p38 (Fig. 5A). Actually, p-T180/Y182 p38 can ensure GSK-3β inactivation by phosphorylating Thr390 (human)/Ser389 (mouse) [41]. At this moment, it has not been well-established that GSK-3β is collectively inactivated by summation of *p*-Ser389 increase and *p*-Ser9 decrease by high concentration of Aβ. Alternatively, the hypothesis that *p*-Tyr216 GSK-3β may phosphorylate Ser422 in Tau at low concentration of Aβ and reduced *p*-Ser9 GSK-3β with an increase of activity may phosphorylate another site of Tau protein at high concentration of Aβ remains to be solved.

It has been disclosed that dephospho-SREBP1 actively functions, and mSREBP1, which is cleaved by SREBP cleavage activating protein (SCAP) is moved to the nucleus, where mSREBP1 plays a role as a transcriptional factor [42]. Indeed, hydrogen peroxide induces lipid biosynthesis through activation of mSREBP1 and ACL expression along with GSK-3β inactivation, leading to an increase of cell mass and cellular senescence [43]. In support to this hypothesis, Aβ peptide was reported to aggravate neuronal senescence in a mouse model of AD [44]. Meanwhile, Aβ oligomerization accelerates senescence in adult hippocampal neural stem and progenitor cells [45].

The ACL up-regulation is irrespective of an increase of *p*-Tyr42 Rho by high concentration of Aβ. Therefore, the function of *p*-Tyr42 Rho under high concentrations of Aβ may be different from that under low concentrations of Aβ. It is notable that *p*-Tyr42 Rho is localized in the nucleus at high concentrations of Aβ (Fig. 6A), where *p*-Tyr42 Rho regulates expression of NAD kinase through its binding to promoter of the *NADK* gene (Fig. 6G), likely leading to an increase of NADP and consequently NADPH levels, which is required as a substrate for fatty acid synthesis or NADPH oxidase. Therein, an increase of cellular mass and cellular senescence through lipid synthesis in response to high concentrations of Aβ may be one of defence mechanisms to reduce superoxide levels through the switch turning down superoxide production induced by NADPH oxidase along with turning on fatty acid synthesis by using NADPH [46]. However, the mechanism by which *p*-Tyr42 RhoA can be translocated to the nucleus in response to high concentrations of Aβ and ROS remains to be discovered. As a defence factor against ROS, nuclear factor erythroid 2-related factor 2 (Nrf2) has been well known to be activated through the dissociation from Keap1 (Kelch ECH associating protein 1) upon ROS. Nrf2 translocates to nucleus, where it binds to antioxidant response element (ARE) and drives the expression of target genes such as heme oxygenase 1 (HMOX1/HO-1), NAD(P)H quinone oxidoreductase 1 (NQO1), glutamate-cysteine ligase (GCL) and glutathione-S-transferase (GST) [47]. Interestingly, neuroprotective curcumin, by inducing Nrf2 and vitagenes including Hsp32 (HO-1/HMOX1), Hsp70 and thioredoxin system and by inhibiting NF-κB activation, prevents neurodegenerative diseases [48,49]. In this study, however, we did not elucidate the relevance of superoxide produced by Aβ to Nrf2.

## 5 Conclusion

We revealed that both high and low concentrations of Aβ are detrimental; while low concentrations of Aβ leads to *p*-Ser422 Tau through activation of RhoA, Src, and Tyr216 GSK-3β (active form), high concentrations of Aβ caused cellular aging through superoxide, p-T180/Y182 p38, *p*-Ser389 GSK-3β (inactive form), mSREBP and ACL activation. In particular, *p*-Tyr42 RhoA is critical for both the elevation of *p*-Ser422 Tau and the expression of ACL and NADK. In particular, *p*-Tyr42 RhoA is localized in nucleus, where regulates expression of specific genes in response to Aβ. In this context, we propose a novel mechanism by which Aβ contributes to neuronal dysfunction through two different signalling pathways of *p*-Ser422 Tau and ACL-mediated neuronal aging depending on Aβ concentrations (Fig. 7G).

## Declaration of competing interest

None.
